# Integrated vector management with the sterile insect technique component for the suppression of *Aedes aegypti* in an urban setting in Indonesia

**DOI:** 10.1371/journal.pntd.0013290

**Published:** 2025-07-07

**Authors:** Hadian Iman Sasmita, Kok-Boon Neoh, Beni Ernawan, Murni Indarwatmi, Indah Arastuti Nasution, Nur Fitrianto, Tri Ramadhani, Tri Isnani, Yorianta Hidayat Sasaerila, Rafa Listyani Rahman, Sri Yusmalinar, Ramadhani Eka Putra, Intan Ahmad, Wu-Chun Tu

**Affiliations:** 1 Department of Entomology, National Chung Hsing University, Taichung, Taiwan; 2 Research Center for Radiation Process Technology, National Research and Innovation Agency (BRIN), Tangerang Selatan, Indonesia; 3 Research Center for Public Health and Nutrition, National Research and Innovation Agency (BRIN), Cibinong, Indonesia; 4 Research Center for Food Technology and Processing, National Research and Innovation Agency (BRIN), Yogyakarta, Indonesia; 5 Department of Biology, Faculty of Science and Technology, University Al Azhar Indonesia, Jakarta, Indonesia; 6 School of Life Sciences and Technology, Institut Teknologi Bandung, Bandung, Indonesia; 7 National Mosquito-Borne Diseases Control Research Center, NHRI, Kaohsiung, Taiwan; Centers for Disease Control and Prevention, UNITED STATES OF AMERICA

## Abstract

**Background:**

Implementing the sterile insect technique (SIT) in areas with high-density target mosquito populations throughout the year is challenging. This study evaluated the effectiveness of releasing radiation-sterilized male *Aedes aegypti* mosquitoes, which were subjected to pre-release control measures in a highly urbanized city.

**Methodology/Principal findings:**

A mark–release–recapture (MRR) trial was conducted to assess the performance of sterile male mosquitoes. The MRR results revealed that the life expectancy of irradiated mosquitoes was 1.2–8.8 days, and that their mean dispersal distance was 60.0–64.3 m. The estimated wild male population ranged from 1,475–2,297 male mosquitoes/ha. In the SIT trial, sterile male *A. aegypti* mosquitoes were released at a rate of 9,000 male mosquitoes/week/ha for 24 weeks. Pre-release control measures, including chemical fogging (Fludora Co-Max EW) and breeding site removal, were employed at the release site. A buffer zone was established by applying residual insecticide (K-Othrine PolyZone SC) and releasing sterile male mosquitoes. In the SIT trial, relative to control sites, the site with sterile male mosquitoes had considerably greater sterility in the field population (greater by 86%), resulting in reductions in the ovitrap density index, and number of wild female mosquitoes captured. In contrast, no significant reduction in ovitrap index was observed. However, despite the gradual recording of low values for egg hatching, ovitrap density index, and female capture, mosquito suppression was incomplete. The mosquito population rebounded shortly after the release of sterile male mosquitoes ended.

**Conclusions/Significance:**

This study underscores the critical role of integrated vector management when the SIT is implemented in highly urbanized areas. It also emphasizes the importance of combining vector control interventions to ensure they are tailored to the geographic context based on logistical feasibility, available local facilities, and local knowledge of the vector.

## Author summary

The sterile insect technique (SIT) involves subjecting laboratory-bred male mosquitoes to radiation, typically gamma rays, X-rays, or electrons, that render them sterile. These sterile male mosquitoes are then released into the field to mate with wild female mosquitoes. From that mating, no viable eggs are produced. The SIT is a mosquito population control strategy that prevents the spread of the dengue virus through *Aedes aegypti* female mosquitoes. Considerable progress has been made regarding the SIT, and its effectiveness has been tested in numerous regions for managing local mosquito populations. In the present study, the field performance of sterile male mosquitoes was evaluated through a mark–release–recapture study, which was followed by an SIT trial. In an SIT pilot trial, pre-release control measures, including insecticide application and mosquito breeding site removal, were applied within the framework of integrated vector management. Community engagement activities were designed to ensure community acceptance and support for the SIT. The trial led to substantial reductions in the egg hatching, numbers of eggs and female mosquitoes despite challenges related to sterile male production and population isolation. This study revealed the key factors contributing to the success of an SIT trial to be the field performance of sterile male mosquitoes, complementary vector control methods, population isolation, and support from local residents.

## Introduction

Controlling the populations of *Aedes aegypti* mosquito, a major vector of arboviruses responsible for life-threatening dengue hemorrhagic fever, is a considerable challenge in many tropical and subtropical countries. Although various dengue vector control programs have been implemented, these programs often do not effectively reduce mosquito populations for several reasons. For example, inappropriate insecticide use causes widespread insecticide resistance [1,2], vectors have plasticity and are able to breed in a wide variety of natural and manmade water-holding containers [3], and low community participation complicates mosquito surveillance and management efforts [4]. Thus, alternative control approaches such as area-wide integrated pest management strategies, including the radiation-based sterile insect technique (SIT) [5], the incompatible insect technique (IIT) based on cytoplasmic incompatibility [6,7], the combination of SIT and IIT [8,9], and the release of insects carrying a dominant lethal [10–12], are gaining popularity in the development of locally tailored integrated vector management strategies [13].

The radiation-based SIT involves the use of optimal doses of ionizing radiation to induce double-stranded DNA breaks that act as dominant-lethal mutations, ultimately inducing sterility in male mosquitoes. The sterile male mosquitoes are then released into the target area to mate with wild fertile female mosquitoes. The eggs produced by the female mosquitoes are not viable, and therefore, the technique can reduce the overall mosquito reproductive success and population growth in the area [14]. Field trials with irradiated sterile *Aedes* male mosquitoes have been performed in several countries [15–21], and the SIT has been integrated with the IIT to ensure successful mosquito suppression with a reduced risk of the accidental release of fertile female mosquitoes into the field [22–25]. Varying levels of success have been achieved using the SIT. For example, the mass release of *A. aegypti* sterile male mosquitoes into a suburb of Havana, Cuba, caused a substantial increase in sterility in the local target mosquito population. In addition, the ovitrap index and the mean number of eggs per trap decreased substantially at 12 and 5 weeks post-release, respectively, and no eggs were identified at the release site after 18 weeks [18]. Bellini et al. [15] reported that releasing sterile male *Aedes albopictus* mosquitoes at the rate of 896–1,590 male mosquitoes/ha/week induced marked sterility and considerably reduced the number of eggs produced by the local population in the Emilia-Romagna region in northern Italy. However, in a pilot study involving sterile *A. albopictus* male mosquitoes released at the rate of 2,500–3,000 mosquitoes/ha/week over 7 weeks in Vravrona, Greece, Balatsos et al. [19] revealed a substantial reduction in the egg hatching at the release site 2 weeks after the first release. However, the number of eggs in the ovitraps was statistically similar to that at the release and control plots [19]. The variation in the literature regarding effectiveness in reducing mosquito populations may be related to differences in the initial population density and the landscape structure of the target sites.

Integrating the release of sterile male mosquitoes with other vector control strategies is recommended to enhance the sustainability and effectiveness of interventions [26,27]. Pre-release control measures, including insecticide fogging and larval source reduction, can effectively suppress the growth of target mosquito populations, resulting in a reduced initial density of mosquitoes [28]. A low initial population allows sterile male mosquitoes to outnumber the wild populations and thus increase the likelihood of mating. In an integrated vector management (IVM) strategy against *A. albopictus* in Mauritius, pyrethroid-based insecticides and source reduction activities led to a reduction in the weekly egg density and number of captured female mosquitoes during the 2 months before the SIT was implemented; these efforts in the initial 2 months sustainably maintained a low mosquito population [16]. Similarly, an IVM approach incorporating breeding site removal and malathion fogging that was performed before the release of irradiated *w*AlbB *A. aegypti* male mosquitoes successfully reduced the target population in a suburban area of Merida, Mexico [25].

Traditionally, dengue vector control strategy in Indonesia has mainly involved chemical interventions and breeding site elimination [29]. However, this strategy has not achieved optimal effectiveness [30], possibly because of the emergence of resistance [31] and challenges in achieving sustained community involvement [4]. Therefore, there is a pressing need to revise the implementation approach of the existing tools. Through the National Strategic Plan, the Indonesian government has emphasized the importance of evaluating novel methods and innovations for dengue control [30], including a field trial implementing the SIT that has been lacking in the past [32]. To date, the radiation-based SIT program in Indonesia has completed the pre-intervention and baseline data collection phase [33–39].

In an SIT program, a mark-release-recapture (MRR) study is a prerequisite before releasing the male mosquitoes. A MRR study can help formulate a release strategy, by assessing the survival and dispersal capacity of sterile male mosquitoes, which are generally compromised due to mass rearing, irradiation, packing, and transporting procedures [40–42], as well as estimating the size of the wild male population at the release site. The MRR procedures involve releasing marked mosquitoes into the field and then subsequently recapturing them at selected time intervals and distances [14,43].

In this study, the MRR procedures [44] were first employed to assess the survival and dispersal of sterile male mosquitoes, as well as to obtain estimates of the abundance of wild male mosquitoes in the target area. The sterile males were released based on the dispersal capacity, survival, and estimated male population in the target area. In particular, owing to their poor dispersal capacity in the highly urbanized target area, we released the sterile males at multiple points along the predefined path. To reduce the initial population density and over-flood the population of wild males, an IVM strategy that combines insecticide application and source reduction was employed with the radiation-based SIT to suppress the *A. aegypti* population in the field.

## Materials and methods

### Ethics statement

The mosquito population intervention designs and protocols in this study were approved by the Review Board of the Research Organization for Nuclear Energy, the National Research and Innovation Agency (NOMOR: B-125/III/TN/3/2022), and the Bandung City government through the Agency of National Unity and Politics (Badan Kesatuan Bangsa dan Politik) (NOMOR: PP.09.01/1481-Kesbangpol/IX/2021 and NOMOR: PP.09.01/867-kesbangpol/V/2022).

### Study area

**Fig 1:**
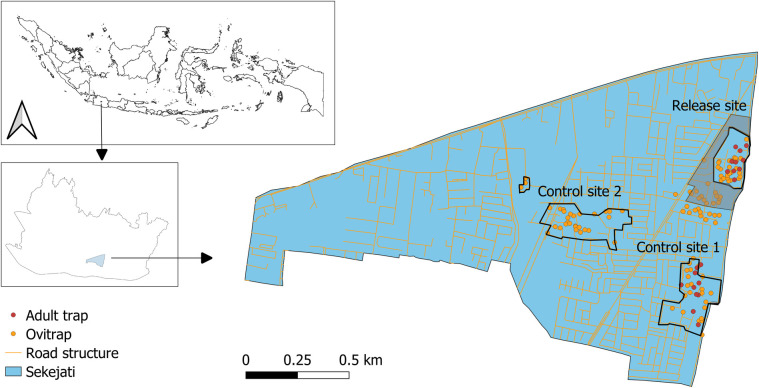
Map of Sekejati urban village in Bandung City, West Java Province, Indonesia. Three locations in the village were used as study sites. RW01 was selected as the release site, while RW04 and RW11 were selected as the control sites 1 and 2, respectively. Dots represent the ovitrap and BG-Sentinel trap distribution. The shaded area represents the buffer zone surrounding the release site. The map base layer was obtained from GADM maps and data (https://geodata.ucdavis.edu/gadm/gadm4.1/shp/gadm41_IDN_shp.zip).

We conducted the trials in highly urbanized areas in Bandung City, Indonesia, where high-density populations of *A. aegypti* are present throughout the year [36]. *Aedes aegypti* was found predominantly in the study sites, both households (98.1% abundance) and public places (86.1% abundance) compared to *A. albopictus* (Households 1.9% and public places 13.9%) [36]. Dengue has been prevalent in Bandung City since 1969–1970 [45]. In 2021, Bandung City recorded 3,743 dengue cases, with 13 being fatal (source: http://data.bandung.go.id/). Sekejati urban village (6°56’48’‘S, 107°39’42’‘E) in the Buahbatu subdistrict of Bandung City was selected as the study site. Sekejati urban village has a tropical climate with distinct dry and wet seasons from June to October and from November to May, respectively. Three neighborhoods in Sekejati urban village, namely RW01 (3.25 ha), RW04 (6.48 ha), and RW11 (6.82 ha), were selected as study plots. The study plots are high-density housing areas, with each house separated by 1–3-m-wide asphalt roads and with a population density of 300–350 inhabitants per ha. In this study, the distances between the neighborhoods were at least 400 m to ensure site independence (Fig 1). The plots are separated from surrounding areas by 2.5- to 4.5-m walls, building structures, 5- to 10-m-wide open sewers, and roads.

### Male production, irradiation, and transportation

**Male mosquito production**. The *A. aegypti* strain used in this study was a local strain from South Tangerang, Indonesia, that has been reared since 2017. The mosquitoes were reared in culture rooms at the Research Center for Radiation Process Technology—National Research and Innovation Agency, Jakarta, at 25.0 ± 1.0 °C, 80.0% ± 2.0% RH, and a 12:12-hour light–dark photoperiod. To obtain adequate eggs for the production of sterile male mosquitoes, adult mosquitoes were reared in large cages (length × width × height = 45 × 45 × 93 cm^3^) at a male:female ratio of 1:1. The adult mosquitoes were provided with a constant supply of 10% sugar solution. Following day 3 post-eclosion, the female mosquitoes were offered a bovine blood meal for an hour a day for 3 consecutive days by using the Hemotek apparatus (Discovery Workshop PS6A/220, Accrington, England) to fuel reproduction. Four days after the first blood feeding, a cup measuring 11 cm in height and 11.5 cm in diameter, intended to serve as an oviposition site, was placed inside the cage for 24 hours. Eggs were harvested by gently pouring the water from the cup through a sieve (fine mesh), and the eggs were then air-dried for 72 hours. Each egg batch was stored in a desiccant chamber. Only the batch in which more than 80% of the eggs hatched was used for irradiation and release experiments.

To produce male pupae for irradiation and release, an amount of eggs equivalent to the density of 3–4 larvae per mL was weighed, and the eggs were hatched in a jar filled with 250 mL of aged tap water [46]. Vitamin C (0.1% w/v) was added to the water to induce hatching [47]. The L1 larvae, together with the hatching solution, were transferred to a plastic tray (length × width × height = 29.5 × 23.0 × 5.0 cm^3^) filled with 1.5 L of aged tap water, and the larvae were fed a 0.5-g mixture of ground dog biscuit (Pedigree, Mars Petcare, Bangkok, Thailand) and locally available chicken liver, which was processed into powder at ratio of 3:1 daily until pupation. The pupae were separated into male and female pupae by using a larval–pupal separator (model 5412) (John W. Hock Company, Gainesville, FL, USA). The male pupae were stored in the larval-rearing tray for 24–48 hours until irradiation.

**Irradiation**. For irradiation, approximately 1,500 male pupae were placed in a plastic container (14 cm in diameter) provisioned with water to ensure a moist condition during the process. A Gammacell 220 irradiator (source: Co-60) (original version: Atomic Energy of Canada, Ottawa, Canada; upgraded version: Izotop, Institute of Isotopes, Budapest, Hungary) was used to irradiate the pupae at a dose of 70 Gy (October 2021: 3495.9 Gy/h, 4806 Ci; November 2022: 3037.2 Gy/h, 4175 Ci; Routine dosimetry: Gafchromic HD-V2 film [Ashland Advanced Materials, Bridgewater NJ, USA]), which induced approximately 98% sterility [33]. After irradiation, the pupae were placed in adult cages measuring 30 × 30 × 30 cm^3^ (BugDorm 1, Mega View Science, Taichung, Taiwan) until adult eclosion. Within 48 hours post-eclosion, the residual female contamination (approximately 0.84%) was thoroughly screened and discarded from all male release batches. Cotton soaked in a 10% sugar solution was introduced as food for the adult mosquitoes.

**Transportation.** The sterile adult male mosquitoes in the adult cages were transferred to customized release cages (length × width × height = 12 × 12 × 15 cm^3^), then the mosquitoes were transported by using a minivan from the rearing and irradiation facilities in South Jakarta to the study sites in Bandung City. The distance between the two places is approximately 167 km, with approximately 3 hours required for transportation. The release cages were covered with wet towels to prevent the loss of humidity inside the cages. Mortality was determined after transport to the study sites. Our previous study revealed that transportation reduced the survival and longevity of sterile mosquitoes but did not affect male mating competitiveness [38].

### Mark–release–recapture trials

For the MRR trials, the sterile male mosquitoes were marked with fluorescent dust (DayGlo Color, Cleveland, OH, USA) following the guidelines for MRR procedures for *Aedes* mosquitoes [44]. Four MRR trials were conducted at the release site: two each with multiple-point release (April 14 2022 and May 26, 2022) and single-point release (April 22, 2022 and June 02, 2022) [48]. Approximately 27,000 and 21,000 color-marked sterile male mosquitoes were released at multiple points and single points, respectively. For the multiple-point releases, equal numbers of male mosquitoes were released at five release points where BG-Sentinel traps (BioGents, Regensburg, Germany) without a lure were positioned. The distances between each release point and the BG-Sentinel traps were 20–60 m. For the single-point releases, the male mosquitoes were released at the central point of the release site, which was 20–100 m from the BG-Sentinel traps (S1 Fig). In the multiple-point release trial, the dyed sterile male mosquitoes were recaptured within the first 3 days after release, while in the single-point release trial, the male mosquitoes were recaptured within the first 6 days after release. Multiple-point release was used to estimate the survival of irradiated mosquitoes and population size of wild male mosquitoes in the area, whereas single-point release was employed to estimate the survival and dispersal capacity of the irradiated mosquitoes [48,49].

### SIT trial with chemical and breeding source reduction interventions

The SIT suppression trial was conducted from May 26, 2022, to November 03, 2022. RW01 was assigned as the release site, while RW04 as control site 1 and RW11 as control site 2. For chemical intervention, space spraying was performed at both the release site and control site 1. Fludora Co-Max 26.3/52.5 EW, a mixture of the active ingredients of flupyradifurone (26.3 g/L) and transfluthrin (52.5 g/L), was sprayed using thermal fogging machines at a dose of 2,350 mL/ha. Space spraying was conducted for 2 consecutive weeks (May 05 and 12, 2022) and was followed by breeding site removal to reduce the initial mosquito population. At the release site, a buffer zone, 2 blocks of residential housing, encompassing 5.84 ha surrounding the release site, was created to prevent the migration of wild mosquitoes from the adjacent areas to the target release site. A residual insecticide, K-Othrine PolyZone 62.5 SC with 6.25% deltamethrin as the active ingredient, was sprayed on the entrances and surrounding walls (2–2.5-m high) of the sites at the rate of 10 mL/L for 25 m^2^ (Fig 1). Additionally, in the buffer zone, approximately 6,000 sterile male mosquitoes were released weekly during the release period. Based on the result obtained from MRR trials, sterile mosquitoes were released along predefined path in the release site (RW01) at a rate of approximately 9,000 male mosquitoes/week/ha on weekly basis for 24 weeks.

### Data collection for entomological parameters

A total of 30 outdoor ovitraps were deployed in each study plot (Fig 1). The number of eggs in each ovitrap was recorded to calculate the ovitrap index (percentage of positive ovitraps per the total number of ovitraps observed) and ovitrap density index (average number of *Aedes* eggs per positive ovitrap). The oviposition stripes containing eggs were air-dried for 3 days to facilitate egg maturation and were then kept in a zip-lock bag for 15 days before being immersed in water to induce hatching. The percentage of eggs that hatched was recorded after 72 hours. On a weekly basis, 10% of the total collected eggs were reared until adulthood to ensure that the prevalence of *A. albopictus* eggs in the outdoor ovitraps was negligible [36]. To sample adult mosquitoes, BG-Sentinel traps (BioGents, Regensburg, Germany) without a lure were set inside the houses of 10 participating residents each in the release site and control site 1. The collected adult mosquitoes were freeze-killed at −20 °C for 30 minutes. The mosquitoes were identified and sexed under a stereomicroscope (Model SMZ 745, Nikon, China). The ovitrap and adult mosquito data were collected on a weekly basis.

### Data analysis

For the MRR, the survival of the released sterile male mosquitoes was estimated by calculating the probability of daily survival, which accounts for average life expectancy. Probability of daily survival is an antilog_10_ of the slope of the regression line from the number of recaptures against the day of recapture [log_10_ (x + 1)] [50], and average life expectancy is 1/-log _e_ (Probability of daily survival) [51]. In this study, a corrected Lincoln Index (P), which accounts for a low recapture rate and compensates for daily survival, was used to estimate the wild male population as follows: P = [R × St (n − m + 1)]/(m + 1), where R is the number of marked male mosquitoes, S is the daily survival rate, t is the sampling day after release, n is the total number of recaptures of both marked and wild adult male mosquitoes, and m is the number of recaptured marked male mosquitoes [52]. The dispersal distance of sterile male mosquitoes was estimated on the basis of the mean distance traveled in annuli (S1 Fig), and the values were corrected using a correction factor to account for unequal trapping densities [48,53,54]. A map of the BG-Sentinel trap placement and virtual annuli was generated in QGIS, version 3.4.15 Madeira, with a background map obtained from GADM maps and data (Creative Commons Attribution-ShareAlike 2.0 license) to calculate the mean distance traveled of the sterile male mosquitoes.

To quantify the effect of the combined sterile male release, chemical, and breeding site removal interventions on mosquito population, we divided the time course into three main periods: the period before mosquito control measures were implemented (pre-intervention period), the period when chemical and breeding site removal were applied (pre-release control measures period), and the period when sterile male mosquitoes were released (release period). The effects of pre-release control measures and sterile male release on egg hatching, ovitrap index, ovitrap density index, and female captured were examined in each time period by comparing release and control sites using generalized linear models (GLMs). In the analysis, to account for temporal structure, weeks were nested within above-mentioned three time periods. We treated site and nested week as fixed effects. We used GLMs with a binomial distribution where the response variables were egg hatching or ovitrap index and a negative binomial distribution (log link) where the response variables were ovitrap density index and females capture. The model included fixed effects for site, the nested week variable, and their interactions. In addition, post-hoc linear contrasts were conducted to compare the release and two controls sites within each week of the three time periods. The analysis allows us to identify site-specific intervention effects at finer temporal resolution.

The relative reduction of measured parameters was calculated as follows: Relative reduction = (1 – odds ratio) × 100%, where the odds ratio is generated from the models. All analyses were conducted using SPSS version 26.0 (SPSS Inc., Chicago, IL, USA) at α = 0.05.

### Community engagement

A total of 42 outreach activities were employed before and during the trials. Community engagement activities were conducted to raise public awareness regarding dengue and mosquito control and to enhance the understanding of SIT-*Aedes* technology for various stakeholders within the local community. The community engagement activities were designed with consideration of the target audience. Initially, public health officials, the local government, and academicians were invited to discuss and provide feedback regarding implementation and regulatory pathways of the planned intervention and communication strategies. We conducted focus group discussions involving local authorities and community leaders, academicians, scientists, local public health officers, and pest control practitioners to discuss, develop, and implement community engagement plan. The feedback received from the stakeholder groups was instrumental in enabling us to tailor our outreach activities to the general public and local community at the study site. We had direct meetings (face-to-face and door-to-door interactions) with residents in the study areas and interactions during a community gathering. Three communication tools were employed to further disseminate information regarding SIT-*Aedes* technology to the general public. An animation video was circulated through chat apps; printed flyers were distributed during direct meetings; and two banners were displayed at the study sites. Four community gathering events were held, and they were attended by approximately 75–100 residents. During the intervention and field releases, briefings regarding the protocol for the SIT were conducted for participating residents (Table 1).

**Table 1:** Community engagement activities conducted before and during interventions.

List of Activities	Target Community	Frequency
Focus Group Discussion	Public health office, local government, and university	2
Direct meeting	Local authorities, head of community, local residents, mosquito surveyors	36
Community gathering event	Local residents	4

## Results

### Mark–release–recapture trials

The percentages of total mosquitoes recaptured during the experiment were 0.28% and 0.43% after multiple-point releases and 0.03% and 0.17% after single-point releases. The probability of daily survival of irradiated mosquitoes ranged from 0.44 to 0.89, and average life expectancy ranged from 1.2 to 8.8 days. Regarding the dispersal of sterile male mosquitoes, the furthest distance at which sterile male mosquitoes were captured was 80 m from the center point of release. Moreover, the mean distance traveled by sterile male mosquitoes ranged from 60.0 to 64.3 m. The population of wild male mosquitoes ranged from 4,793–7,466 or was equivalent to 1,475–2,297 male mosquitoes/ha, respectively (Table 2).

**Table 2:** Mark–release–recapture trials with multi-point and single-point release at the release site.

Parameters	Multi-point release (green)	
	MRR trial 1	MRR trial 2
Date	April 14, 2022	May 26, 2022
No. male released	12,000	15,000
Total recapture (%)	0.28	0.43
Probability of daily survival	0.63	0.44
Average life expectancy (days)	2.20	1.21
Estimated population (Corrected Lincoln Index)	4793.16	7465.99
	**Single-point release (blue)**	
	**MRR trial 1**	**MRR trial 2**
Date	April 22, 2022	June 02, 2022
No. male released	9,000	12,000
Total recapture (%)	0.03	0.17
Probability of daily survival	0.89	0.68
Average life expectancy (days)	8.81	2.56
Mean distance traveled (m)	60.0	64.3

### SIT intervention

**Fig 2:**
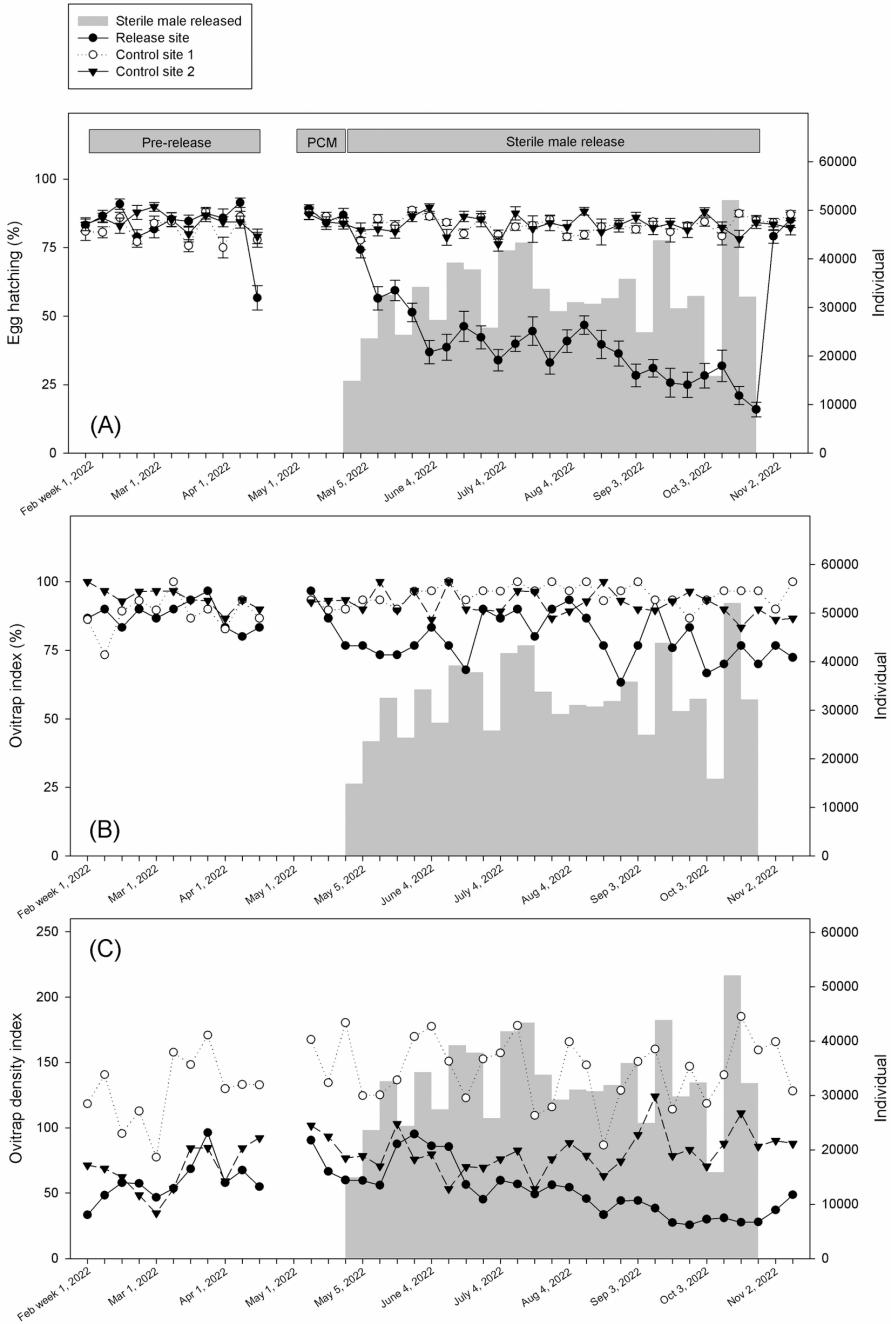
Weekly egg hatching (±SE) (A), ovitrap index (B), and ovitrap density index (C) in pre-intervention, pre-release control measures, and sterile male release periods. Vertical grey area represents number of sterile male mosquitoes released.

**Fig 3:**
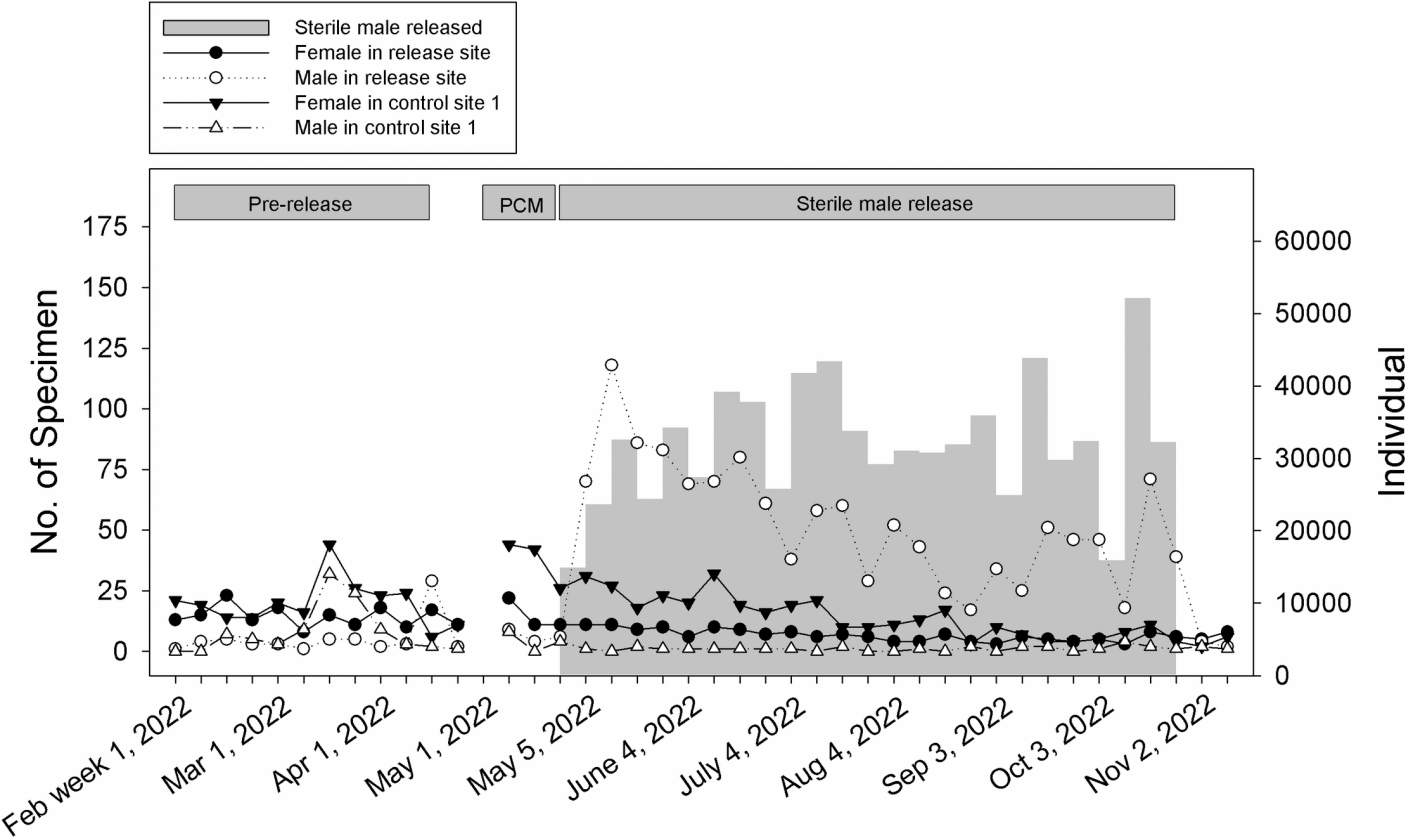
Weekly number of *Aedes aegypti* adults collected using BG-Sentinel traps throughout the study period. Vertical grey area represents number of sterile male mosquitoes released.

A total of 768,723 sterile male mosquitoes were released at a rate of 15,000–52,000 male mosquitoes per week (Figs 2 and 3). The percentage of egg hatching significantly declined at the release site compared to those at control site 1 and control site 2 by 86.1% and 86.3%, respectively, during the release period (Table 3). The reduction in egg hatching at the release site was dependent on the week of release (release vs control site 1: Wald χ^2 ^= 110.355, P < 0.01; release vs control site 2: Wald χ^2 ^= 106.225, P < 0.01) (S1 Table). In particular, post-hoc analysis revealed that a significant reduction in egg hatching was observed in the 3^rd^ week after release and continued to remain low in the next 12 weeks compared to control sites 1 and 2. The lowest percentage of egg hatching at 16.0% ± 2.6% was recorded in week 1 of November 2022 (Fig 2A).

**Table 3:** Results of the generalized linear model of the egg hatching, ovitrap index, ovitrap density index, and number of females captured at the release site compared with those at the control sites (reference) during the pre-intervention, pre-release control measures and release periods.

Parameters	Periods	Coefficient (B)	S.E.	Statistical values	P value	Exp(B) (CI 95%)	RR (%)
*Release site vs Control site 1*							
**Egg hatching**	Pre-intervention	0.195	0.107	3.302	0.069	1.215 (0.985 - 1.500)	–
	Pre-release control measures	-0.044	0.295	0.022	0.883	0.957 (0.537 - 1.707)	–
	Release	-1.972	0.067	863.232	< 0.001	0.139 (0.122 - 0.159)	86.1
**Ovitrap index**	Pre-intervention	-0.115	0.215	0.288	0.592	0.891 (0.585 - 1.358)	–
	Pre-release control measures	0.374	0.616	0.367	0.545	1.453 (0.434 - 4.863)	–
	Release	-1.008	0.746	1.824	0.177	0.365 (0.085 - 1.576)	–
**Ovitrap density index**	Pre-intervention	-0.862	0.075	131.446	< 0.001	0.422 (0.365 - 0.489)	57.8
	Pre-release control measures	-0.719	0.184	15.346	< 0.001	0.487 (0.340 - 0.698)	51.3
	Release	-1.218	0.052	545.503	< 0.001	0.296 (0.267 - 0.328)	70.4
**No. female captured**							
	Pre-intervention	-0.365	0.1	13.209	< 0.001	0.694 (0.570 - 0.845)	30.6
	Pre-release control measures	-0.414	0.206	4.024	0.045	0.661 (0.441 - 0.991)	33.9
	Release	-0.775	0.093	69.902	< 0.001	0.461 (0.384 - 0.552)	53.1
*Release site vs Control site 2*							
**Egg hatching**	Pre-intervention	0.054	0.11	0.244	0.621	1.056 (0.851 - 1.309)	–
	Pre-release control measures	0.083	0.288	0.083	0.773	1.087 (0.617 - 1.912)	–
	Release	-1.989	0.067	872.014	< 0.001	0.137 (0.120 - 0.156)	86.3
**Ovitrap index**	Pre-intervention	-0.045	0.212	0.045	0.832	0.956 (0.631 - 1.449)	–
	Pre-release control measures	0.904	0.574	2.029	0.115	2.469 (0.802 - 7.607)	–
	Release	-0.19	0.129	2.174	0.14	0.827 (0.643 - 1.064)	–
**Ovitrap density index**	Pre-intervention	-0.331	0.075	19.364	< 0.001	0.718 (0.619 - 0.832)	28.2
	Pre-release control measures	-0.262	0.184	2.03	0.154	0.770 (0.537 - 1.103)	–
	Release	-0.488	0.052	87.379	< 0.001	0.614 (0.554 - 0.680)	38.6

For ovitrap index, no significant reduction was observed between release and control sites 1 and 2 over the experimental periods (Table 3). Before the release, ovitrap density index at the release site was 51.3-57.8% and 28.2%, significantly less than that at control site 1 and 2, respectively (Table 3). During the release period, the ovitrap density index at the release site decreased by 70.4% compared to the control site 1, and by 38.6% compared to release site 2 (Table 3). The effect of male release on ovitrap density index at the release sites depends on week of release (release vs control site 1: Wald χ^2 ^= 53.437, P < 0.01; release vs control site 2: Wald χ^2 ^= 36.811, P < 0.05) (S1 Table). For instance, a significant reduction in ovitrap density index was observed in the 1^st^ week and 17^th^ week of release. Significant difference in the number of wild female mosquitoes captured at the release site compared to control site 1 during pre-intervention and pre-release control measures (Table 3). However, overall, the number of female captured was further reduced from 33.9% to 53.1% during the release period compared to the control site 1 (Table 3).

## Discussion

Site selection is crucial when a SIT pilot trial is implemented through the release of mass-reared, sterile male mosquitoes. Many researchers select sites where the target vector population is isolated from external influencing factors, including geography, climate, mosquito biology, human activities, or a combination of these factors [55–57]. *Aedes aegypti* mosquitoes thrive in urban cities. With their rapid urbanization and development of large and unplanned populations, Indonesian cities provide numerous oviposition sites for mosquitoes and abundant human hosts for blood meals. However, achieving geographical isolation in urban settings can be extremely challenging. In the current study, we implemented a strategy to address this challenge. We established buffer zones and deployed chemical barriers around release sites to prevent mosquito migration. Furthermore, we applied IVM approaches, such as insecticide spraying and weekly breeding source removal, to reduce the size of the initial mosquito population before introducing sterile male mosquitoes. Our results clearly demonstrate a marked reduction in egg hatching as well as a decrease in the ovitrap density index and number of female mosquitoes captured during the release of sterile mosquitoes. However, it is worth noting that the effectiveness of these barriers was limited; the mosquito population rebounded shortly after we terminated the release.

The success of the release of sterile male mosquitoes hinges on a deliberate strategy of over-flooding, which increases the likelihood of these male mosquitoes finding compatible female mates. Over-flooding strategy can be achieved by either increasing the frequency of releases per week or by including a larger number of sterile mosquitoes in each release. For example, with the implementation of a weekly sterile male release at a frequency of 2–3 times, a suppression trial against *A. albopictus* mosquitoes in Spain achieved a remarkable reduction of 70% to 80% in the numbers of eggs and adult female mosquitoes collected [21]. In the present MRR trial, we observed that the life expectancy of the male mosquitoes averaged at 3.70 ± 3.46 days, and the estimated male population varied from 1,475–2,297 male mosquitoes/ha. We employed the strategy of releasing sterile male mosquitoes at a rate of approximately 9,000 male mosquitoes/ha, with the number of sterile male mosquitoes being 4- to 6-fold higher than that of wild male mosquitoes, and this release was conducted on a weekly basis. Our release rate was suboptimal given the high prevalence of dengue and the high mosquito density in the study area [36]. However, achieving the ideal 10:1 ratio with twice-weekly releases [25,26,49] proved unattainable due to logistic challenges, including manpower and transportation constraints. Furthermore, the excessive presence of sterile male mosquitoes in residential areas may be perceived to be a nuisance by some residents. Therefore, achieving a balance between effective over-flooding and community acceptance in terms of the number of sterile male mosquitoes released remains a key challenge in our efforts to control mosquito populations.

In the present study, we adopted a comprehensive approach that combined the SIT with an IVM strategy to address the challenges associated with over-flooding. Our aim was to reduce the initial mosquito population density before introducing sterile male mosquitoes. To achieve this, we implemented two key measures: thermal fogging using a mixture of transfluthrin and flupyradifurone (Fludora Co-Max EW) and the reduction of mosquito breeding sources. The insecticide mixture was reported to be effective against wild pyrethroid-resistant *A. aegypti* [58]. Statistical analysis demonstrated that the implementation of the pre-release control measures did not lead to a considerable reduction in the ovitrap index, ovitrap density index, and female mosquito population at the release site when compared to pre-intervention periods (Table 3). However, it is worth noting that a sharp decline of ovitrap index and ovitrap density index was observed two weeks later after pre-release control measures (Fig 2B and 2C). The result indicates a delayed but significant effect of pre-release control measures in reducing the initial mosquito population before the sterile male release.

Pre-release control measures involving thermal fogging and mosquito breeding source reduction can increase the ratio of released mosquitoes to wild mosquitoes in the environment. The present study demonstrated that the egg hatching was reduced by approximately 86%. We also observed substantial reductions in other measured parameters. The ovitrap density index and number of female mosquitoes captured decreased considerably compared with those at the control sites. These findings are further supported by the previous study that a minimum 40% - 50% egg hatching reduction is required to cause a decrease in the ovitrap index, ovitrap density index, and the number of female mosquitoes captured [15,59].

Natural barriers that can be used to isolate target mosquito populations in urban settings are rare. In the present study, several manmade structures, including roads, sewers, and buildings near the release site, impeded mosquito migration from the surrounding environment. To further enhance this isolation, we established a buffer zone by releasing 6,000 sterile male mosquitoes per week, and we implemented a chemical barrier by using K-Othrine PolyZone, a long-lasting residual spray. These artificial barriers appeared, to a certain extent, to have effectively prevented mosquitoes in the surrounding environment from dispersing into the target sites. This observation is supported by our findings regarding the entomological parameters, with consistently low values recorded throughout the 24 weeks of sterile male mosquito release. By contrast, Tur et al. [21], who combined the SIT with Bti (*Bacillus thuringiensis israelensis*), larvicide, and lethal oviposition traps, reported a small reduction in the induced egg sterility of *A. albopictus*. This small reduction, despite large reductions having been noted in the numbers of female mosquitoes and eggs, indicates that induced sterility may have confounded mosquito migration from nearby areas in their study.

Our findings reveal that despite the present study gradually recording low values for egg hatching, mosquito suppression was incomplete. Furthermore, the mosquito population rebounded shortly after we terminated the release of sterile male mosquitoes. However, Gato et al. [18] conducted a study in Cuba involving the release of irradiated male mosquitoes, and they achieved 100% induced egg sterility, resulting in complete mosquito suppression, with no eggs collected after 17 weeks of release. Mosquito elimination continued for at least 4 weeks, even after release was discontinued. The field trial in Cuba was conducted in the southwestern suburb of Havana, where the study sites were surrounded by nonresidential areas, including forests, rivers, agricultural land, a railway, and a national highway. The findings of Gato et al. [18] and the present study indicate that the artificial barriers in our study may have incompletely prevented mosquito dispersal from the surrounding areas into our target sites. Furthermore, the residual effect of the chemical barrier spray used in our study typically lasts for approximately 90 days, depending on local weather conditions. We suspect that the reduced residual effect of the spray over time may have compromised the effectiveness of our chemical barriers.

In conclusion, the implementation of the SIT in large, unplanned cities in Indonesia presents major challenges because achieving complete mosquito population isolation is often impractical in highly urbanized areas. The present study represents a pioneering effort to demonstrate that the introduction of a chemical barrier through long-lasting residual insecticide spraying and the establishment of buffer zones in the vicinity of a target area can prevent mosquito migration into the area. Logistical and facility constraints pose a challenge in the inundation of sterile male mosquitoes. To address the challenge of a high *Aedes* mosquito population density, integrating the SIT, which involves the release of sterile male mosquitoes, with pre-release control measures, including chemical fogging and breeding source reduction, is essential. These interventions considerably reduce the initial mosquito population and yield satisfactory results. This study indicates the critical role of IVM in the SIT in highly urbanized areas. It emphasizes the importance of implementing various vector control interventions, either individually or in combination with other interventions, that are tailored to the specific geographic context and that are developed with consideration of logistical feasibility, local facilities, local knowledge regarding the vector, and local acceptance of the SIT.

## Supporting information

S1 Fig Placement of BG-Sentinel traps in study plots. Virtual concentric lines at radii of 20, 40, 60, 80, and 100 m from the release point are presented. Map base layer was obtained from GADM maps and data (https://geodata.ucdavis.edu/gadm/gadm4.1/shp/gadm41_IDN_shp.zip).
(TIF)

S1 Table SIT data for *A. aegypti* control in Indonesia. (XLSX)
