# Supplementary figures and images for: Integrated vector management with the sterile insect technique component for the suppression of Aedes aegypti in an urban setting in Indonesia
Source: PLoS Negl Trop Dis. 2025 Jul 7;19(7):e0013290. doi: 10.1371/journal.pntd.0013290 (PMC12244676; doi:10.1371/journal.pntd.0013290)

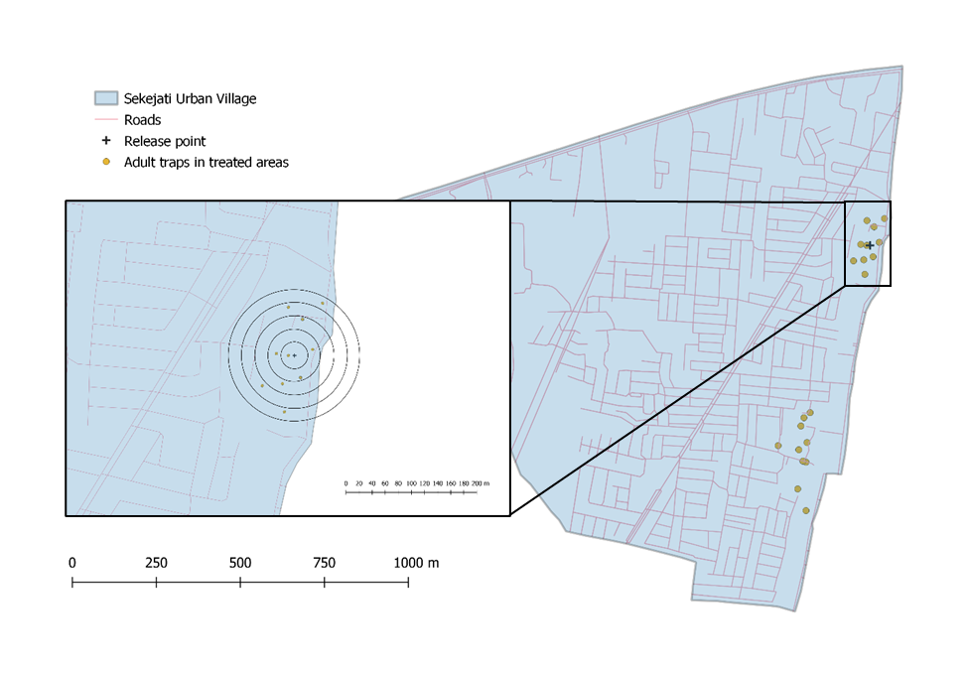

Supplement: S1 Fig — Virtual concentric lines at radii of 20, 40, 60, 80, and 100 m from the release point are presented. Map base layer was obtained from GADM maps and data (https://geodata.ucdavis.edu/gadm/gadm4.1/shp/gadm41_IDN_shp.zip). (TIF) [file pntd.0013290.s001.tif]
